# A rapid, high-throughput method for determining chronological lifespan in budding yeast

**DOI:** 10.14440/jbm.2018.272

**Published:** 2018-12-18

**Authors:** Zachery R. Belak, Troy Harkness, Christopher H. Eskiw

**Affiliations:** 1 Department of Food and Bioproduct Sciences, College of Agriculture and Bioresources, University of Saskatchewan, Saskatoon, SaskatchewanS7N 5A8, Canada; 2 Department of Biochemistry, Microbiology and Immunology (BMI), College of Medicine, University of Saskatchewan, Saskatoon, SaskatchewanS7N 5A8, Canada

**Keywords:** MTT assay, *Saccharomyces cerevisiae*, caloric restriction, chronological lifespan

## Abstract

The budding yeast *Saccharomyces cerevisiae* is a major model system in the study of aging. Like metazoans, yeast lifespan is extended by caloric restriction and treatment with pharmacological agents which extend lifespan. A major workhorse of aging research in budding yeast is the chronological lifespan assay. Traditionally, chronological lifespan assays consist of taking regular samples of aging yeast cultures, plating out aliquots on agar, and counting the resulting colonies. This method, while highly reliable, is labor-intensive and expensive in terms of materials consumed. Here, we report a novel MTT-based method for assessing chronological lifespan in yeast. We show that this method is equal to the colony counting method in its rigorous and reliable measurement of lifespan extension in yeast as a result of caloric restriction, and is able to distinguish known long-lived and short-lived yeast strains. We have further developed this method into a high-throughput assay that allows rapid screening of potential anti-aging compounds as well as yeast strains with altered lifespan. Application of this method permits the rapid identification of anti-aging activities in yeast and may facilitate identification of materials with therapeutic potential for higher animals and, most importantly, humans.

## INTRODUCTION

The budding yeast *Saccharomyces cerevisiae* is a prominent model system in modern biology, particularly for the study of aging and lifespan [1-5]. Yeast combines advantages such as low cost, ease of genetic manipulation, and rapid generation time, as well as possessing high similarity to metazoans in its response to factors that affect lifespan and the evolutionarily conserved nature of genes that influence longevity [2-4]. Increased lifespan in eukaryotes is induced by caloric restriction (CR) and pharmacological agents which are thought to modulate cellular nutrient sensing [6-8]. Caloric restriction is defined as a reduction of an organism’s available nutritional energy without inducing malnutrition [6]. In the case of yeast, this means reduction, or alteration of the concentration of carbon source used for energy production without reducing availability of amino acids, vitamins, or inorganic nitrogen sources to inadequate levels [5,8-10]. Pharmacological agents can also extend lifespan by mimicking the effects of CR by disrupting the cell’s ability to “sense” nutrients within their environment. These agents include rapamycin and metformin [6,11,12]. Rapamycin disrupts nutrient sensing by inhibition of the TOR kinase [12]. Metformin is hypothesized to inhibit the mitochondrial respiratory chain, with the net effect of reducing cellular energy production (increased AMP/ATP ratios), and therefore is also a mimetic of CR [6,11]. In addition to these well-characterized CR mimetics, the dietary polyphenols (-)-epigallocatechin gallate (EGCG) and resveratrol have been shown to extend lifespan in metazoan model systems including *C. elegans* and *D. melanogaster* [6]. These compounds are hypothesized to extend lifespan through both antioxidant activity and modulation of nutrient sensing pathways *via* activation of Sirtuin proteins [6]. Resveratrol and rapamycin have been shown previously to extend lifespan in yeast [13-15]. However, neither EGCG nor metformin has been previously investigated for lifespan extension in this model system.

Lifespan in budding yeast is represented by two metrics: replicative life span (RLS) —the number of daughter cells produced over the lifetime of a given mother cell; and chronological lifespan (CLS) —the length of time non-dividing cells survival [1,6,17]. CLS is assayed by growing yeast cells to stationary phase followed by periodic removal, dilution, and agar plating of aliquots of the aging culture [1]. The resultant colony forming units (CFUs) are then used to determine survival curves. This is known as the colony counting (CC) chronological lifespan assay. While the introduction of automated methods for CC have increased the efficiency of this method somewhat, the preparation of potentially hundreds of agar plates and the required dilutions and plating are a significant burden in terms of time and resources, and this methodology is very poorly suited to high-throughput applications. Powers *et al*. used decreases in optical density of yeast cells growing in 96-well plates to assess lifespan and this method led to the global identification of yeast mutants that alter CLS [15]. The method relies on the gradual decrease in optical density in aging cultures due to autolysis of dead cells. This technique suffers from the disadvantage that it does not distinguish living cells from intact or fragmented dead cells. Furthermore, decreases in optical density were marginal at best and can take up to weeks to appear significant. We therefore sought to design a new method for assessing chronological lifespan in budding yeast that would be inexpensive, rapid, accurate, and amenable to high-throughput screening of potential anti-aging compounds such as those described above.

One of the most widely used methods for assessing eukaryotic cell number are absorbance-based assays based on the conversion by viable cells of a tetrazolium compound to a corresponding highly colored formazan product [18,19]. The most common embodiment of these assays relies on the reduction of yellow 3-(4,5-dimethylthiazol-2-yl)-2,5-diphenyltetrazolium bromide (MTT) to a purple-colored formazan product with an absorption peak at 550 nm [18]. In mammalian cells, MTT is reduced to the formazan by a number of mitochondrial NAD(P)H oxioreductase enzymes, and under defined conditions the increase in absorbance of the formazan product at 550 nm is proportional to the number of viable cells [19]. The MTT assay has also been applied to the determination of cell number of simple eukaryotes such as fungi, protozoans, and yeasts, as well as in prokaryotic cells [19]. Previous work has reported the use of MTT reduction as an indicator of viability in yeasts in the absence of menadione [20-22]. However, it appears that many yeast species or strains are unable to efficiently reduce MTT and addition of menadione as an intermediate electron acceptor is required for efficient MTT reduction [19,23,24]. Jahn and colleagues report a simple, high-throughput colorimetric assay for measuring cell concentration of the pathogenic yeast Candida albicans in the context of antifungal susceptibility testing involving addition of 0.5 mg/ml MTT and 0.1 mM menadione to actively growing cultures [24]. Separately, Hodgson and colleagues reported that MTT was only effectively reduced by actively growing *S. cerevisiae* cells and formazan formation was absent in cultures of stationary phase cells, leading to the conclusion that MTT assays are only valuable for proliferating cells [22]. In this publication, we report the development of a novel yeast rapid chronological lifespan (RCL) assay based on these previously reported MTT-based assays. We show that in the presence of menadione and glucose the assay accurately reflects viable cell number in aging yeast cultures. The assay is used to demonstrate the lifespan increasing effect of caloric restriction and accurately distinguishes between known long-lived and short-lived yeast strains. Finally, we describe a high-throughput RCL (HTRCL) assay which permits rapid screening of compounds, yeast mutants, or both, for alterations in lifespan. These assays represent a substantial improvement in cost, time investment, and capability over previous chronological lifespan assays in budding yeast.

## MATERIALS AND METHODS

### Reagents, media, and yeast strains

Yeast extract, peptone, yeast nitrogen base without amino acids, sodium chloride, sodium dihydrogen phosphate, disodium hydrogen phosphate, dimethyl sulfoxide (DMSO), and glucose were Fisher^®^ Bioreagent^®^ grade, purchased from ThermoFisher Scientific Inc. (Fairlawn, NJ, USA). 2-methyl-1,4-naphthoquinone (menadione) and MTT were acquired from Acros Organics^®^ (New Jersey, NJ, USA). NP-40, 2-propanol (HPLC grade), resveratrol, and (-)-epigallocatechin gallate (EGCG) were obtained from Sigma-Aldrich^®^ (St. Louis, MO, USA). ACS grade hydrochloric acid was purchased from Anachemia Canada Co. (Montreal, QC, Canada). Metformin and rapamycin were supplied by Santa Cruz Biotechnology Inc. (Dallas, TX, USA). Strains of *S. cerevisiae* used were WT (*MATa ade2 his3 leu2 lys2 ura3*, [25]), *fob1Δ (MATa his3 leu2 met15 ura3 fob1Δ::kanMX6*, [25]), *apc10Δ* (*MATa his3 leu2 met15 ura3 apc10Δ::kanMX6*, [26]), *apc5*^CA^ (*MATa ade2 his3 leu2 ura3 apc5*^CA^*-PA::His5*, [27]), *apc5*^CA^ *apc10Δ* (*MATa his3 leu2 met15 ura3 apc5*^CA^*-PA::His5 apc10Δ::kanMX6*, [26]), and *sch9Δ* (*MATa ade2 his3 leu2 lys2 ura3 sch9Δ::His5*, this study). Yeast extract peptone dextrose (YPD) (1% w/v yeast extract, 2% w/v peptone, 2% w/v glucose), and complete minimal media (CM; 0.67% w/v yeast nitrogen base without amino acids, 0.02% w/v complete amino acid mixture) were used as described previously [28]. Cell concentrations in yeast cultures were assessed by dilution of culture 1/100 in PBS (140 mM NaCl, 10 mM sodium phosphate pH 7.4) and cell number counted using a Beckman-Coulter Multisizer^®^ 3 Coulter counter. All spectrophotometric measurements were made with a BioRad Smartspec^®^ plus spectrophotometer. All error bars in the included figures represent the standard error of three independent replicates.

### Dilution/plating based chronological lifespan assays

Yeast was grown overnight at 30°C in YPD, collected by centrifugation (1500 × G, 5 min, RT), washed in sterile water, centrifuged, and resuspended at a concentration of 1 × 10^7^ cells/ml in CM media containing either 2% w/v or 0.2% w/v glucose as indicated. Where applicable, compounds such as rapamycin were added to cultures during the exponential growth phase, before cell reached stationary phase. Cultures were then incubated at 30°C with shaking for 48 h to allow cells to reach stationary phase, after which cultures began to age and the number of viable cells began to decrease [1,28]. Cultures were subsequently maintained at 30°C with shaking for the duration of the lifespan experiments. At the indicated time points, 100 μl of the culture was removed, diluted 1/10000 with distilled water, and 100 μl of the resulting dilution plated on YPD agar plates in triplicate for technical replicates. After 48 h incubation at 30°C plates were scanned using a standard commercially available document scanner (Epson Perfection^®^ V300 Photo) and colonies counted using the OpenCFU software [29]. To ensure accuracy, some plates were manually counted and in all cases the number of colonies was within 1% of the value predicted by the OpenCFU software. Data presented is the average of three independent biological replicates.

### Analysis of optimal assay conditions for MTT-based chronological lifespan assays

Yeast was grown to stationary phase (72 h) at 30°C in YPD, collected by centrifugation (1500 × G, 5 min, RT), washed in sterile water, centrifuged, and resuspended at a concentration of 1 × 10^7^ cells/ml in sterile water. For experiments monitoring the rate of formazan product accumulation and the effect of glucose and menadione addition, 2.5 ml of the yeast suspension was combined with 2.5 ml of a solution containing 1 mg/ml of MTT plus the indicated additives (0.2 mM menadione, 4% w/v glucose, or both) and incubated at 30°C with shaking. At the indicated time points, 250 μl of the mixture was removed, combined with 250 μl of solubilization solution (50% v/v 2-propanol, 0.2% NP-40, 20 mM hydrochloric acid), and placed on an agitator at room temperature for 30 min. The resulting solution was then centrifuged (15000 × G, 5 min, RT) and the absorbance of the resulting supernatants measured at a wavelength of 550 nm. For assessing the accumulation of formazan product in relation to cell concentration the stationary phase cells obtained as above were resuspended in sterile water at concentrations of 1 × 10^7^, 5 × 10^6^, 2.5 × 10^6^ or 1.25 × 10^6^ cells/ml in a volume of 2.5 ml, combined with 2.5 ml of a solution of 4% glucose, 1 mg/ml MTT and 0.2 mM menadione, and treated exactly as described above.

### Rapid chronological lifespan assay

RCL Reagent Solution contained 4% w/v glucose, 1 mg/ml MTT, and 0.2 mM menadione and was filter sterilized and stored at **−**20°C prior to use. Yeast was cultured overnight at 30°C in YPD, collected by centrifugation (1500 × G, 5 min, RT), washed in sterile water, centrifuged, and resuspended at a concentration of 1 × 10^7^ cells/ml in CM media containing either 2% w/v or 0.2% w/v glucose as indicated. Cultures were then incubated at 30°C with shaking for 48 h prior to, and for the duration of, the lifespan experiments. At the indicated time points, 250 μl of the culture was removed and combined with an equal volume (250 μl) of RCL Assay Solution and mixed. The mixture was then incubated with shaking at 30°C for 4 h. An equal volume (500 μl) of solubilization solution (50% v/v isopropanol, 0.2% NP-40, 20 mM hydrochloric acid) was then added and the mixture placed on an agitator at room temperature for 30 min. The resulting solution was then centrifuged (15000 × G, 5 min, RT) and the absorbance of the resulting supernatants measured at a wavelength of 550 nm. Where indicated, the student’s *t*-test was used to compare the average% initial absorbance at 550 nm to the average% initial cell number as determined by the CC assay.

### High throughput rapid chronological lifespan assay

For application of the RCL assay to 96-well plate format, yeast cells were grown overnight in YPD at 30°C. Cells were then collected by centrifugation (1500 × G, 5 min, RT), washed once in sterile water, again collected by centrifugation, and suspended in CM medium containing 2% w/v glucose at a concentration of 1 × 10^7^ cells/ml. The resulting suspension was aliquoted into 96-well plates (200 μl per well) and a range of concentrations of the indicated pharmacological agents added in the form of a solution in DMSO or water. DMSO or water was added to control wells such that the concentration of vehicle was identical in all wells of the plate. Plates were then incubated at 30°C for 48 h prior to beginning the assay. This further ensured that the cells were undergoing exponential growth in the presence of specific drugs prior to HTRCL assays being performed. After 24 h, the plates were mixed to ensure even suspension of the cells and 50 μl was removed into the wells of a fresh plate. To this replicate plate, 50 μl per well of RCL Reagent Solution (see above) was added and the plate mixed. Plates were incubated for 4 h at 30°C, 100 μl per well of solubilization solution (see above) was added, and plates placed on an agitator at room temperature for 30 min. Finally, the absorbance of each well of the plate at 550 nm was determined using a Packard^®^ Spectracount^®^ model BS10001 plate reader/spectrophotometer.

## RESULTS

### Determination of optimal conditions for RCL assay

**Figure 1.**
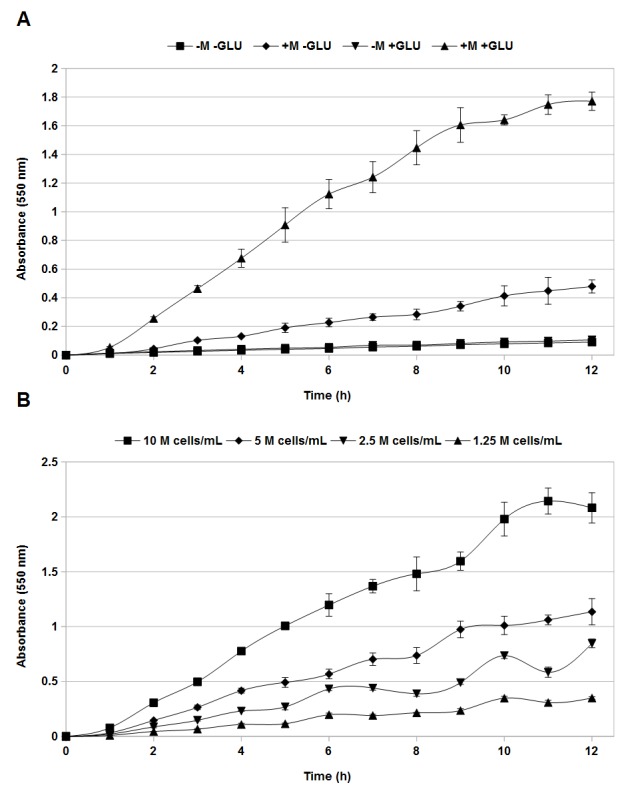
Determination of optimal rapid chronological lifespan assay conditions. Stationary phase aging yeast cultures were combined with MTT-containing assay solution and incubated. At the indicated times aliquots were removed, combined with solubilization reagent, and absorbance determined at 550 nm. **A.** Accumulation of formazan product in the presence or of menadione (+/-M) and glucose (+/-GLU). **B.** Accumulation of formazan product in presence of both menadione and glucose in the assay medium as a function of cell concentration (M = 10^6^). Error bars represent the standard error of three independent replicates.

Our first step in developing an MTT-based yeast chronological lifespan assay was to investigate optimal conditions for formazan product formation in aging yeast cultures. Aging cells from stationary phase cultures were isolated by centrifugation, washed, resuspended in water, and employed in assays as described. It has been noted previously that some yeast strains are not able to directly reduce MTT to formazan product and addition of menadione is required for this transformation [19,23,24]. Treatment of aging yeast cultures with MTT in the absence of menadione resulted in no appreciable accumulation of formazan product even after 12 h of incubation (**Fig. 1A**). Upon addition of MTT in the presence of 0.1 mM menadione, we observed a relatively linear increase in absorbance corresponding to formation of the formazan product (**Fig. 1A**). However, the increase in absorbance was gradual, with an absorbance of 0.479 ± 0.046 being achieved after 12 h of incubation. Formation of formazan product is dependent on cellular metabolic activity [19]. As aging cultures become depleted in glucose and other nutrients [1], we hypothesized that supplementation of cultures with glucose during the assay would increase production of formazan product. Indeed, addition of 2% w/v glucose to the MTT assay mixture dramatically increased the rate at which formazan product accumulated, presumably by permitting living cells to utilize mitochondrial function required for formation of formazan product. These data show that optimum product formation in aging yeast cultures is achieved in the presence of menadione and glucose, and therefore these materials were included in the reagent in all subsequent experiments.

**Figure 2.**
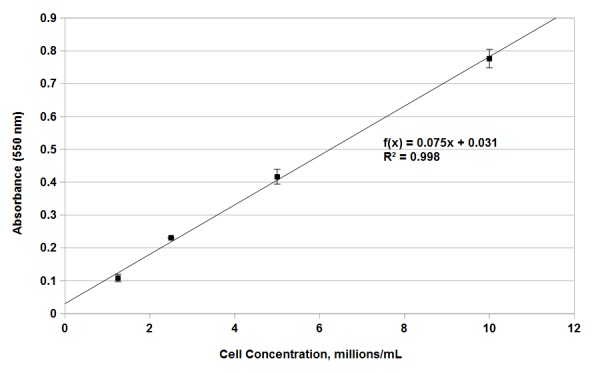
Correlation between cell number and absorbance at 550 nm. Stationary phase aging yeast cultures were combined with RCL assay reagent containing menadione and glucose, incubated for 4 h, combined with solubilization reagent, and absorbance determined at 550 nm. Mean absorbance is plotted on the *y*-axis against cell concentration on the *x*-axis. Error bars represent the standard error of three independent replicates.

Our next goal was to confirm the linear relation between formazan product formation and the number of viable cells present. Stationary phase yeast cells were resuspended in water at concentrations of 1 × 10^7^, 5 × 10^6^, 2.5 × 10^6^ and 1.25 × 10^6^ cells/ml, combined with RCL reagent, and the accumulation of formazan product followed over 12 h of incubation (**Fig. 1B**). Accumulation of product was essentially linear from one to six hours of incubation. Between 6 and 12 h, absorbance values began to fluctuate and become non-linear, possibly due to metabolic disruption of cells due to accumulated formazan product (**Fig. 1B**). Based on the results of these experiments, we established four hours as the optimal incubation time at which concentration of the formazan product was most linearly related to cell concentration. This relationship is demonstrated in **Figure 2** where absorbance is plotted against cell number for mixtures incubated with the MTT assay solution for 4 h. The relation is highly linear over the range examined with an R2 value of 0.998 (**Fig. 2**). The data presented in **Figure 1** and **Figure 2** confirm that menadione is required for conversion of MTT to the colored formazan product as reported previously [23,24] and that addition of glucose substantially increases the rate of formazan product formation. Finally, the results show that four hours is the optimal time for incubation with MTT solution to provide the most accurate estimation of cell number. We therefore utilized a 4 h incubation in a solution containing final concentrations of 2% w/v glucose, 0.5 mg/ml MTT, and 0.1 mM menadione for all subsequent experiments.

### RCL assays exhibit a relationship between signal and viable cell number over time and demonstrate extended lifespan under conditions of caloric restriction

**Figure 3.**
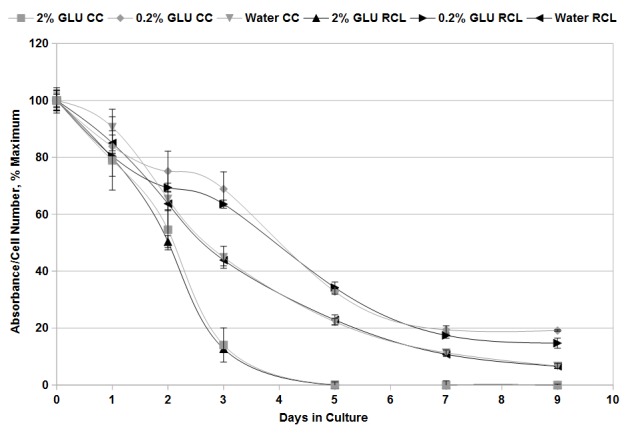
Analysis of yeast chronological lifespan under conditions of caloric restriction. Stationary phase aging yeast cultures in CM media containing either 2% or 0.2% glucose (GLU), or of cells suspended in water, were incubated for the indicated number of days (*x*-axis). Each day, an aliquot was removed from the culture and subjected to either the traditional CC assay (grey) or the novel RCL assay described here (black). For CC assays, viability is expressed as a percent of the mean cell number at day 0, for RCL assays as a percent of the mean absorbance at day 0 (*y*-axis). Error bars represent the standard error of three independent replicates.

Previously, the established method for determination of chronological lifespan in budding yeast has been to plate a known dilution of the aging yeast culture and count the resulting colonies [1], herein referred to as the CC method. Our next step in development of the RCL assay was to demonstrate that our method accurately reported the proportion of viable cells over time *via* comparison of RCL data with data obtained using the classic CC method (**Fig. 3** and **Fig. S1**). Results of CC assays are typically represented as the percent of viable cells remaining after a given period as compared to the initial maximal number of cells [1] and so we similarly chose to express the results of the RCL assay as the percent absorbance at a given time relative to the absorbance at the initial maximum. Initially, we compared the decrease in viable cell number over time of wild-type yeast grown in rich (2% w/v glucose) medium using the CC and RCL assays (**Fig. 3** and **Fig. S1**). We found a very high degree of correlation between the two assays, with percent viability decreasing to 78.9% ± 5.03% (CC) and 79.8% ± 1.4% (RCL) at 1 d 54.5% ± 5.6% (CC) and 50.4% ± 2.1% (RCL) at 2 d, 14.0% ± 2.9% (CC) and 12.7% ± 0.7% (RCL) at 3 d, and finally to 0.11% ± 0.01% (CC) and 0% (RCL, no difference in absorbance from blank after 4 h) after 5 d of incubation (**Fig. 3** and **Fig. S1**). These data demonstrate that the RCL assay gives highly comparable values to the CC assay within the measured experimental error.

One of the most powerful factors in increasing lifespan in budding yeast and metazoan organisms is caloric restriction (CR) [4,6-10]. Since reduction of glucose concentration has been shown to substantially prolong yeast chronological lifespan [5,9,10], we assessed the ability of the RCL assay to accurately report cell viability over time under conditions of caloric restriction. Comparison between CC and RCL assays of calorie restricted yeast showed consistent values obtained by the classic CC method and our new RCL assay (**Fig. 3** and **Fig. S1**). These data demonstrate the effectiveness of the RCL assay for accurately reporting viable cell number over time in aging yeast cultures and its ability to demonstrate extended chronological lifespan of yeast subjected to caloric restriction.

### RCL assay demonstrates increased or decreased lifespan of known long- or short-lived yeast mutants

**Figure 4.**
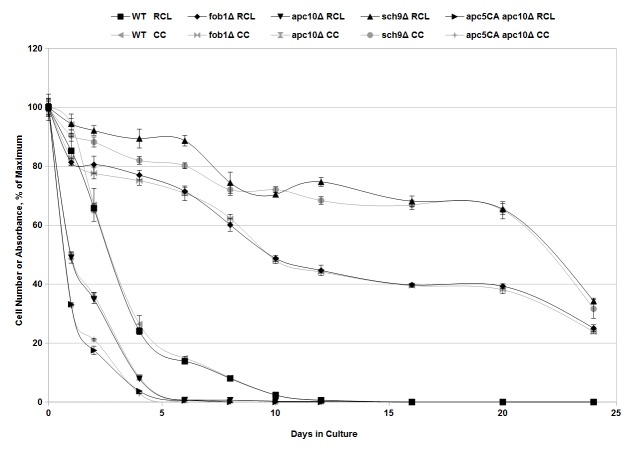
Analysis of long-lived and short-lived yeast mutants. Stationary phase aging cultures of various known long-lived or short-lived yeast strains were prepared in CM containing 2% glucose were incubated for the indicated number of days (*x*-axis). Each day, and aliquot was removed from the culture and subjected to either the traditional CC assay (grey) or the novel RCL assay described here (black). For CC assays, viability is expressed as a percent of the mean cell number at day 0, for RCL assays as a percent of the mean absorbance at day 0 (*y*-axis). Strains are identified above the panel. Error bars represent the standard error of three independent replicates.

One of the advantages of budding yeast as a model system is the ability to easily create and isolate mutant strains in order to better understand the biochemical pathways that underlie a variety of processes including aging [2-4]. Over time, a number of yeast mutants with abnormally long or short chronological lifespans have been identified and characterized [15,17,27,28,30,31]. These include cells lacking *FOB1* or *SCH9*, which confer long life, or cells expressing the short-lived *apc10Δ* or *apc5*^CA^ apc10*Δ* mutations [17,30,31]. While deletion of *FOB1* has been previously shown to increase replicative lifespan [30], Here we show that it also increases CLS (**Fig. 4** and **Fig. S2**). We further established the efficacy of the RCL assay as a measurement of chronological lifespan in yeast by applying it alongside the CC assay of aging cultures of the above yeast strains (**Fig. 4** and **Fig. S2**). There was very high agreement between the proportion of viable cells as determined by the CC assay and the RCL assay (**Fig. 4** and **Fig. S2**). In comparing percent viability obtained by the CC assay versus the RCL assay, all values corresponded within measured experimental error, except that the RCL assay reported slightly higher values for *sch9Δ* cells at 1, 2, 4, 6, and 12 days while the RCL assay reported a slightly lower value than the CC assay for *apc5*^CA^ *apc10Δ* cells at the 2-day time point (**Fig. 4** and **Fig. S2**). These slight deviations, while greater in magnitude than the experimental errors and therefore significant from a statistical point of view (student’s *t*-test), did not affect the ability of the assay to accurately distinguish long-lived from short-lived yeast strains and may represent slight metabolic differences between stains. Overall, these experiments show that the RCL assay is effective for analysis of yeast mutants with altered lifespan.

### HTRCL assay demonstrates increased lifespan of yeast treated with known pharmacological enhancers of lifespan

One of our primary motivators in developing the RCL assay was to devise a method for the rapid screening of pharmacological compounds with the ability to increase lifespan. A number of previously identified compounds that have been shown to extend lifespan in metazoans have also been shown to extend chronological lifespan in budding yeast [6,12-15]. Given the advantages of yeast for studies of aging, we utilized it as a model for the rapid screening of compounds with potential anti-aging activity. To achieve this, we adapted the RCL assay to a high-throughput format using 96 well plates. In this assay, two candidate compounds can be screened for effects on chronological lifespan at ten different concentrations simultaneously on a single plate Briefly, a plate containing the yeast strain of interest and the compound of interest at varying concentrations is incubated to the point of stationary phase. Then, a portion of the mixture from each well is transferred to a second plate and the RCL performed, and finally, 48 h later the mixture is subjected to the RCL assay a second time. This gives, for each set of four wells, the proportion of viable cells after 48 h relative to the initial value and is therefore an indicator of increased or decreased chronological lifespan. The above observation that numbers of wild-type cells in rich media were decreased to approximately 50% after 48 h (**Fig. 3** and **Fig. S1**) led us to choose this time point for analysis in the HTRCL assay. Although we did not observe an alteration in the rate of formazan product formation by yeast cells in the presence of the pharmacological agents we tested (data not shown), it is possible that the presence of these compounds or others could alter this process. To normalize these effects, the quantity of formazan produced at the initial time point in the presence of the added agent was compared against the quantity produced in the same wells 48 h later, any effect of a given compound on formazan product formation is cancelled, leaving only the difference in viable cell number in the result.

**Figure 5.**
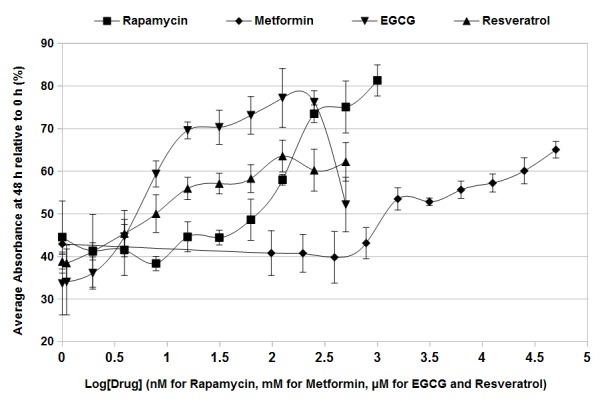
Analysis of anti-aging compounds by the high-throughput rapid chronological lifespan assay. A 96-well plate containing wild type yeast in CM media containing 2% glucose and the indicated concentrations of either rapamycin, metformin, EGCG, or resveratrol, was incubated to the point of stationary phase (0 h). A portion of the mixture from each well was transferred to a second plate, and an equal volume of RCL added followed by 4 h incubation. Solubilization reagent was then added to the wells of the replicate plate and the absorbance of each well at 550 nm determined. After 48 h further incubation the process was repeated. For each set of wells, the mean absorbance at 48 h was divided by the mean absorbance at 0 h to derive the mean% absorbance relative to the initial value (*y*-axis). The concentrations of the agents used are plotted in log_10_ scale on the *x*-axis. Units are nM for Rapamycin and μM for Metformin, EGCG, and Resveratrol. Error bars represent the standard error of three independent replicates.

To demonstrate the usefulness of the HTRCL, we treated wild-type cells in rich media with four different pharmacological agents known to extend chronological lifespan in metazoans: the macrolide mTOR inhibitor rapamycin [6,12,14,15] the anti-diabetic drug metformin [1,2]; and the dietary polyphenolic compounds EGCG and resveratrol [6,13]. The results show that in all four examples viability of cells treated with vehicle alone had decreased to between 30% and 45% at 48 h (**Fig. 5**), consistent with what was observed in RCL assays performed above (**Fig. 3** and **Fig. S1**). Treatment with rapamycin at concentrations above 125 nM caused increased cell viability after 48 h with a maximum of 81.3% ± 3.7% viability retained at a concentration of 1 μM (**Fig. 5**), consistent with previously published data [14,15]. Metformin led to increased 48 h viability at concentrations from 3.125 mM to 50 mM (**Fig. 5**), with maximum retention of viability of 65.1% ± 1.9% at 50 mM (**Fig. 5**). To our knowledge, this is the first demonstration of enhanced chronological lifespan in budding yeast treated with metformin. The well-studied polyphenol EGCG increased the 48 h viability of aging yeast cells at concentrations of 7.8 μM to 500 μM (**Fig. 5**). Maximum 48 h viability of 77.2% ± 6.9% was observed at 125 μM EGCG, while viability decreased slightly at higher concentrations to 52.2% ± 6.4% at 500 μM (**Fig. 5**). Another well-studied dietary polyphenol, resveratrol, also increased 48 h cell viability in aging cells at concentrations above 7.8 μM, with maximal effect of 63.6% ± 3.7% viability observed at a concentration of 125 μM (**Fig. 5**). While resveratrol has been shown previously to extend lifespan in yeast at similar concentrations [13], so far as we are aware these results constitute the first demonstration of increased lifespan in yeast in response to EGCG. These results demonstrate that the HTRCL assay is effective as a tool for initial screening of lifespan extending compounds in the budding yeast model system.

## DISCUSSION

One of the most important methods for analyzing the effects of genetic, nutritional, and pharmacologic modifiers of the aging process in yeast is the chronological lifespan assay [1,28]. Previously, chronological lifespan assays have been conducted using the CC method, whereby aliquots of aging yeast cultures are diluted and plated on agar and the resulting colonies counted to determine the decrease in viable cells over time [1]. Here we describe RCL and HTRCL assays based on the conversion of MTT to a colored formazan product which are rapid, inexpensive, and highly accurate.

MTT-based assays have been applied previously to determine viable cell number in exponentially growing cultures of yeast, but they have not previously been applied to aging yeast cells [21,22,24]. The data presented in **Figure 1** and **Figure 2** show that this assay can be effectively extended to measurement of yeast cell numbers in aging cultures, providing a method for rapidly assessing yeast chronological lifespan. Our data demonstrates that, in the presence of menadione and glucose, production of formazan product by aging yeast cells is linear in relation to cell number (**Fig. 2**). We have also demonstrated that the novel RCL assay is able to accurately demonstrate the previously observed extension in yeast chronological lifespan due to caloric restriction (**Fig. 3** and **Fig. S1**). The RCL assay is also equal to CC assays in its ability to distinguish yeast mutants with abnormally short or long lifespans (**Fig. 4** and **Fig. S2**). We have further developed this basic RCL assay into a high-throughput method using 96-well plates (HTRCL) that permits rapid screening of pharmacological compounds with potential anti-aging effects (**Fig. 5**). The HTRCL assay can also be adapted to rapid screening for changes in chronological lifespan of yeast mutants or transformants, alone or in combination with pharmacological modifiers of lifespan. Taken together, the data presented demonstrate that the RCL and HTRCL assays are equivalent to traditional CC assays in their ability to accurately measure chronological lifespan in yeast with the added benefits of increased economy, reduced time input, and the capacity for high-throughput analysis.

In general, the RCL assay showed slightly less experimental error than CC assays and less variability between biological replicates. This is likely due to the reduced number of manipulations involved, including dilution steps and spreading of yeast suspensions on agar plates. A limitation of the RCL assay is that it was unable to detect very small numbers of viable cells. For example, after 5 d in culture in 2% w/v glucose wild-type cells showed a decrease in viability to 0.11% ± 0.01% by CC assay but the RCL assay showed no difference from blanks containing no cells (**Fig. 5**). This limitation is unlikely to be problematic since in assessing chronological lifespan it is the overall rate of decrease in cell viability with time that is of interest and not the ability to determine the exact point at which complete loss of viability of the culture is achieved [1]. However, in those cases where it is desirable to determine viability of very small numbers of cells or where it is necessary to determine the exact point at which complete viability is lost the RCL assay may not be appropriate. With respect to the HTRCL assay, we did consider that the presence of various pharmacological agents could alter the rate of formazan product accumulation, particularly where the compound being tested has the potential to modify or lower the metabolic activity of cells. We addressed this concern through the design of the assay by comparing the quantity of formazan product produced in the presence of the test compound at 0 h to the quantity produced in the same wells 48 h later. In this way, any subtle effects of the compound or vehicle on MTT metabolism will be compensated for to the greatest extent possible. Furthermore, other methods used to assess cell proliferation, such as trypan blue or thymidine analogue incorporation, result in the same survival curves as MTT. We would also emphasize that the HTRCL is meant primarily as a screening tool for the identification of compounds and the relevant concentrations of interest and that any promising compounds would be subjected to more detailed investigation using the RCL or CC assays. This point is underlined by our observations of the effect of EGCG on cell viability (**Fig. 5**). We noted that the 48 h viability increased at concentrations of 7.8 μM to 125 μM and then decreased at concentrations of 250 μM to 500 μM.

These observations reinforce the utility of the HTRCL assay as a screening tool for establishing the useful range of concentrations of candidate compounds for further study, and avoiding either concentrations too low to be effective or so high as to cause toxicity. We would also underline that both the RCL and HTRCL assays are highly versatile and that the temperature of incubation (30°C), the time of incubation in the presence of MTT (4 h), or the time between the initial and final measurements in the HTRCL assay (48 h), can be easily altered to suit the needs of particular yeast strains or experimental requirements.

In conclusion, the RCL and HTRCL assays described here represent novel methods for the analysis of chronological lifespan and rapid screening of potential anti-aging compounds in budding yeast. The assays described here exhibit high precision and accuracy, and are more economical, more rapid, and more amicable to analysis of larger numbers of samples in parallel. While an optical density-based, high-throughput method for discovery of long-lived yeast strains has been previously described [15] the previous assay suffers from low sensitivity, does not distinguish living from dead cells, and requires weeks. The HTRCL assay described here constitutes a very rapid, accurate, and high-throughput method for screening compounds with potential anti-aging effects in yeast, or indeed in any model organism. The ability of the HTRCL assay to rapidly screen for lifespan extending activity promises the potential for identification of numerous novel anti-aging compounds. Given the abundance of previous data showing that caloric restriction as well as many compounds known to extend lifespan in metazoans also extend chronological lifespan in yeast [6,8-10,13-15], it is hoped that the ability to rapidly identify anti-aging activity in yeast will facilitate identification of materials with therapeutic potential for higher animals and, most importantly, humans.

## Supplementary Material

Supplementary information **Figure S1.** Analysis of yeast chronological lifespan under conditions of caloric restriction, log scale.
**Figure S2.** Analysis of long-lived and short-lived yeast mutants, log scale.
Supplementary information of this article can be found online at
http://www.jbmethods.org/jbm/rt/suppFiles/272.
